# Knowledge, beliefs and practices regarding osteoporosis among female medical school entrants in Pakistan

**DOI:** 10.1186/s12930-017-0036-4

**Published:** 2017-09-18

**Authors:** Muhammad Bilal, Abdul Haseeb, Aleena Zehra Merchant, Abdur Rehman, Mohammad Hussham Arshad, Maarij Malik, Asad Hammad Ur Rehman, Pallavi Rani, Emaan Farhan, Taha S. Rehman, Umer Sultan Shamsi, Sadia Aminah

**Affiliations:** 1 Dow University of Health Sciences, Karachi, Pakistan; 2 Aga Khan University Hospital, Karachi, Pakistan; 3 Karachi Grammar School, Karachi, Pakistan; 4 The Lyceum School, Karachi, Pakistan

**Keywords:** Osteoporosis Knowledge, Risk factors, Practices, Pakistan, Medical students

## Abstract

**Background:**

Osteoporosis is a growing health problem around the world. The increasing incidence of osteoporotic fractures coupled with the lack of knowledge about the disease in the general community means that the disease is continuously increasing the burden on health sector and the general population. The purpose of the study is to assess knowledge, attitudes and practices about osteoporosis among female medical school entrants in Karachi.

**Methods:**

This is a descriptive cross-sectional study conducted amongst 400 female medical school entrants of DOW University of Health Sciences (DUHS) and Jinnah Sindh Medical University (JSMU). A pre validated questionnaire, Osteoporosis Knowledge Assessment Tool (OKAT), was distributed amongst the participants. A food frequency questionnaire was also distributed to determine calcium intake. Descriptive statistics and Chi square test were used to compare the two groups of students with SPSS (20.0) being utilized for analysis. A p value of <0.05 was considered as significant.

**Results:**

The mean age of the participants was 19.4 ± 1.2 years. Only 8.0% of the participants had a good score pertaining to knowledge about osteoporosis whereas majority of the participants (49.0%) had a poor score. Perceived susceptibility was low as only 14.0% of the participants believed that they were at a high risk for osteoporosis. The RDA for calcium was equal to or greater than 700 mg per day which was met by only 29.0% of the participants despite of the high motivation towards consuming a calcium rich diet. Exercise levels were insufficient in terms of both, duration and the recommended type of exercise. Only 12.0% of the participants engaged in exercises according to the recommended guidelines. Moreover, only 5.5% subjects were involved in definitive behaviors to improve bone health.

**Conclusions:**

Participants possessed an insufficient knowledge of the disease and that too was not adequately applied in preventative practices. There is a serious lack of adoption of preventative practices for osteoporosis. This was primarily due to little appreciation of the seriousness of osteoporosis. Hence, this study highlights the dire need for awareness about practices and attitudes related to the disease. Furthermore, it could be of paramount importance to future studies conducted on practices and beliefs related to osteoporosis.

## Background

Osteoporosis is a major health concern around the world. The disease results in porous bones causing reduction in bone density and strength increasing the risk of fractures [1]. Worldwide, osteoporosis causes more than 8.9 million fractures annually, resulting in an osteoporotic fracture every 3 s [2]. The disease can affect both sexes but females are at a higher risk of developing the disease [3]. Hence, there is a high burden of disease due to its higher prevalence amongst women. There is limited data on the prevalence of osteoporosis in Pakistan. Young and premenopausal Pakistani women diagnosed with osteopenia also have a likelihood to develop osteoporosis [4].

Knowledge about osteoporosis primarily focuses upon the risk factors for the disease and the extent to which this knowledge is utilized to take preventative measures constitutes the practices of the study population. The attitude of study participants towards the disease is mainly dependent on their beliefs related to it, such as perceived susceptibility.

The modifiable risk factors for osteoporosis include inadequate dietary intake of calcium and vitamin D, consumption of carbonated drinks and an inactive lifestyle [5]. Menopause, aging and genetic predisposition are amongst the non-modifiable risk factors for the disease [5]. Preventing osteoporosis requires strategies which aim to increase bone density during the early years of life [6]. Some of the methods include physical exercise, adequate intake of calcium and vitamin D as well as abstinence from smoking [7]. Knowledge about osteoporosis is also essential in prevention of the disease. Various studies have been conducted with regard to the knowledge and beliefs about osteoporosis amongst women. Insufficient knowledge of osteoporosis was found amongst women aged above 25 years in a study conducted in United States [8]. The dietary consumption of calcium in Asian women residing in Australia was low (less than 800 mg a day) and they possessed limited information about osteoporosis [9].

The increasing occurrence of osteoporosis in the Asian continent has also escalated health expenditures on the disease [10]. In Asia, osteoporosis is often left undiagnosed and untreated [2]. This is true for even those patients who have the highest susceptibility to the disease and have also suffered fractures previously. Furthermore, this issue is more severe in rural settlements. A large proportion of the population resides in rural areas in countries such as China and India. People in rural settlements tend to treat hip fractures at home rather than opting for hospital management and care [2]. In Pakistan, osteoporosis is a pressing health issue because of severe nutritional deficiencies as well as a lack of sufficient diagnostic means [2].

Moreover, very few studies have been conducted in Pakistan with regard to osteoporosis and these have concluded that although women possessed awareness about osteoporosis, the knowledge didn’t translate into practices for preventing the disease [11]. However, with regard to osteoporosis awareness amongst students, current literature is limited with only a small study being conducted amongst students in Quetta [10]. This calls for further studies on a broader scale using a variety of parameters. It is imperative to understand that a sound knowledge about osteoporosis and its prevention amongst students is significant as students can convey knowledge to the general population. Osteoporosis preventative programs amongst students (young women) lowers the susceptibility to the disease in the older age [12]. This is because maintenance of bone strength and mass in young age lowers the risk for the disease in later years of life. Development of future preventative strategies require assessment of knowledge about osteoporosis and the current practice of its prevention amongst young women [13].

Considering the scarcity of data in our country in general, and the student community in particular, the aim of this study was to assess the knowledge, beliefs and practices of osteoporosis among female medical school entrants in Karachi, Pakistan.

## Methodology

This cross-sectional study was conducted amongst the female medical school entrants in two different universities in Karachi, namely DOW University of Health Sciences (DUHS) and Jinnah Sindh Medical University (JSMU). The study was population based and was carried out in January 2016 over a period of 4 weeks. A convenience sample of 400 students (200 students from each university) was selected for the study. This sample size was calculated on the assumption that 50% of medical students possess knowledge of osteoporosis. Furthermore, an open EPI calculator at 95% confidence interval was used which yielded a sample of 384. However, for statistical convenience we recruited 400 subjects. The students from each university were selected as participants on the basis of convenient accessibility and proximity to the researcher. Participants selected for the study didn’t have a previous history of specific diseases (for instance malabsorption syndrome, osteomalacia and rickets) and weren’t a part of any previous studies pertaining to osteoporosis information. However, people excluded from the study were those who had been identified with osteoporosis, were not 1st year medical students, or were unable to communicate in English.

A pre-tested and pre-validated questionnaire was delivered amongst the study population after verbal consent. The study subjects were interviewed using a validated questionnaire, Osteoporosis Knowledge Assessment Tool (OKAT) [14]. This tool includes 20 questions to determine knowledge about osteoporosis. The tool is based on 4 basic knowledge areas about osteoporosis which include possible risk factors, preventative strategies, identification of the disease and treatment availability. Beliefs about osteoporosis were assessed using the Osteoporosis Health Belief Scale (OHBS) [15]. It is comprised of 42 questions which pertain to different aspects such as realization about vulnerability to osteoporosis, appreciation about severity of the disease, perception about barriers to calcium intake and exercise and health awareness. The 42 items are divided into seven subscales each consisting of 6 items. It is a 5 point Likert scale and its format includes included “strongly agree, agree, disagree, strongly disagree and undecided”.

Practices regarding osteoporosis were determined using a questionnaire which was comprised of questions on favorable and unfavorable behaviors towards osteoporosis. The favorable behaviors tested were calcium consumption in diet, exercise and sunlight exposure. The unfavorable practices assessed included smoking and alcohol consumption. Moreover, the questionnaire also included an adjusted and tested 40 response frequency food questionnaire to determine the dietary calcium consumption amongst the participants [16].

The study was performed after approval from the Institutional Review Board of Dow University of Health Sciences (DUHS).

### Data analysis

The variables which were collected were categorized as knowledge, beliefs and practices. To compare the two groups of students, descriptive statistics and Chi squared test were used. SPSS (20.0) was used to analyze the information. A p value of <0.05 was considered as significant.

### Scoring methods for questionnaires

The portion pertaining to knowledge had the following score criteria: 1 point for a correct answer and 0 point for a Do Not Know or an incorrect answer. The total score out of 20 was multiplied by 5 in order to generate a total out of 100. The criteria was set as follows: <20: very poor, 20–40: Poor, 41–60: Average, 61–85: Good and 86 or more: very good. The questionnaire to determine practices related to osteoporosis had been pre coded to determine the favorable and unfavorable behaviors. Calcium consumption was calculated from the data obtained from the food frequency questionnaire.

The portions of each food consumed per week by each participant was obtained and their calcium value was calculated from the food reference information. This was used to determine the weekly calcium intake of each subject in the study. Further calculation revealed the daily calcium intake for each student. The total daily calcium intake was then classified as sufficient (equal to or more than 700 mg per day) and insufficient (less than 700 mg per day) according to the RDAs for calcium in South-East Asia [17]. Assessment of physical activity was made by time period and type of exercises performed during the week.

## Results

The mean age of female medical school entrants was 19.4 ± 1.2 years. Based on our thresholds for the knowledge section in this survey, 196 (49.0%) subjects had an average score, 164 (41.0%) had a poor score, and 6 (1.5%) had a very poor score. Only 32 (8.0%) participants had a good score. Only 2 of our study participants had a score above 85. Mean and median scores were 33.2 ± 8 and 34, out of 100, respectively.

Table 1 depicts the responses obtained on knowledge about osteoporosis risk factors and preventive practices. It was evaluated that family history of osteoporosis was considered as a risk factor by 144 (36.0%) subjects. Surprisingly, old age as a risk factor was not appreciated by 150 (37.5%) participants, whereas premature menopause and smoking was considered as a causative agent by 21 (5.3%) and 60 (15.0%) participants respectively. Moreover, students of DMC had greater knowledge of the preventive impact of weight bearing exercise than students from JSMU (130 vs 50; p < 0.01). It was also evident that only 188 (47.0%) were aware about dietary sources of calcium, and the students from JSMU had a significantly better knowledge than DMC students in this area (126 vs 62; p < 0.01). Only a small number of participants (22.0%) considered osteoporosis as an asymptomatic medical disease.

**Table 1:** Depicts the responses obtained on knowledge about osteoporosis risk factors and preventive practices

Risk factors/preventive practices	Total (N = 400)	Dow Medical College (N = 200)	Jinnah Sindh Medical College (N = 200)	p value
Family history of osteoporosis	144 (36.0)	80 (40.0)	64 (32.0)	0.17
Female sex	370 (92.5)	188 (94.0)	182 (91.0)	0.51
Old age	250 (62.5)	130 (65.0)	120 (60.0)	0.15
Premature menopause	21 (5.3)	12 (6.0)	9 (4.5)	0.19
Smoking	60 (15.0)	36 (18.0)	24 (12.0)	0.14
Physical activity (weight bearing exercises)	180 (45.0)	130 (65.0)	50 (25.0)	*<0.01
Daily calcium requirement from food	92 (23.0)	50 (25.0)	42 (21.0)	0.66
Sources of calcium	94 (23.5)	62 (31.0)	126 (63.0)	*<0.01
Hormone therapy after menopause	102 (25.5)	48 (24.0)	54 (27.0)	0.39

Table 2 illustrates beliefs of our participants regarding osteoporosis. It was revealed that only 56 (14.0%) subjects believed that their chance of getting osteoporosis are high. With regards to perception towards calcium consumption and its advantages, it was demonstrated that 126 (31.5%) subjects felt that calcium rich food is difficult to eat, 37 (9.3%) females revealed that they disliked it or were unable to tolerate a calcium rich diet and a similar number of participants (N = 37) believed that calcium containing items are expensive. While evaluating our study subjects’ concern about osteoporosis, 222 (55.5%) stated that if they developed osteoporosis it would change their lives, and a similar number of participants (N = 222) revealed that osteoporosis discussion scares them. 324 (81.0%) believed that suffering from this disease would make their daily activities challenging.

**Table 2:** Illustrates beliefs of our participants regarding osteoporosis

Perceived susceptibility	Total (N = 400)	Dow Medical College (N = 200)	Jinnah Sindh Medical College (N = 200)	p value
Chances of getting osteoporosis are high	56 (14.0)	32 (16.0)	24 (12.0)	0.18
We are more likely to get the disease	74 (18.5)	42 (21.0)	32 (16.0)	0.23
Family history makes us more likely to get osteoporosis	32 (8.0)	22 (11.0)	10 (5.0)	<0.01
Perceptions, towards barriers to calcium intake				
Eating calcium rich food is difficult	126 (31.5)	70 (35.0)	56 (28.0)	0.17
Calcium rich foods do not agree with us or we dislike calcium rich foods	37 (9.3)	17 (8.5)	21 (10.5)	0.45
Calcium rich foods are too expensive	37 (9.3)	15 (7.5)	22 (11.0)	0.88
Perceptions, towards benefits of calcium intake				
Eating calcium rich foods reduces risks of broken bones	334 (83.5)	162 (81.0)	172 (86.0)	0.88
Eating calcium rich foods helps to build bones	269 (67.3)	141 (70.5)	128 (64.0)	0.22
Eating calcium rich foods prevents future problems from osteoporosis	332 (83.0)	168 (84.0)	164 (82.0)	0.79
Perceived seriousness of osteoporosis				
If we had osteoporosis it would change our whole life	222 (55.5)	120 (60.0)	102 (51.0)	0.31
Thought of osteoporosis scares us	222 (55.5)	119 (59.5)	103 (51.5)	0.39
Having osteoporosis would make daily activities more difficult	324 (81.0)	168 (84.0)	156 (78.0)	0.22
Health motivation towards osteoporosis				
We are motivated frequently do things to improve our health	248 (62.0)	156 (78.0)	92 (46.0)	<0.01
We are motivated to eat a well-balanced diet	243 (60.8)	152 (76.0)	91 (45.5)	<0.01
Motivated to exercise regularly	91 (22.8)	66 (33.0)	25 (12.5)	<0.01

When participants’ health motivation towards osteoporosis was queried, 62% (N = 248) revealed that they would carry out steps to improve their health, and 60.8% (N = 243) expressed motivation to follow a well-balanced diet. Only 22.8% (N = 91) were motivated enough to exercise regularly. In comparison with JSMU students, DMC students revealed better health motivation towards osteoporosis (p <  0.01). Despite this, their motivation to exercise regularly was poor.

The average calcium consumption of our study subjects was 510 mg/day and only 116 (29.0%) attained the RDA for calcium. Among the subjects who attained the RDA, 40 (34.5%) were taking multivitamin tablets and 60 (51.7%) were not consuming any kind of mineral supplements. In relation to intake of the top calcium providing items, our study participants responded for milk, yoghurt, cheese, sea food and rice products. Table 3 demonstrates the duration of weight bearing exercise per week carried out by our participants. It was found that only 48 (12.0%) participants fulfilled recommended guidelines for type and duration of exercise. The commonest mode of exercise among female students was walking. Moreover, 304 (76.0%) participants responded that they expose themselves to the sun for at least 30 min per week. The majority of our subjects (N = 395) were teetotalers and non-smokers. Furthermore, only 22 (5.5%) subjects were involved in definitive behaviors to develop healthy bones and 70 (17.5%) subjects intended to regularly involve themselves in such practices.

**Table 3:** Demonstrates the duration of weight bearing exercise per week carried out by female medical students

Duration	Total (N = 400)	Dow Medical College(N = 200)	Jinnah Sindh Medical College(N = 200)	p value
<30 min/week	172 (43.0)	90 (45.0)	82 (41.0)	0.22
30–60 min (1 h)	102 (25.5)	36 (18.0)	66 (33.0)	<0.01
60–90 min	78 (18.8)	44 (22.0)	34 (17.0)	0.31
>90 min	48 (12.0)	30 (15.0)	18 (9.0)	0.04

## Discussion

The objective of the study was to determine the knowledge, beliefs and practices concerning osteoporosis of female medical school entrants in Karachi, Pakistan. The purpose for selecting medical school entrants stems from the fact that they have not been taught about osteoporosis at the undergraduate level and all of their knowledge would be from high school studies in Pakistan. Knowledge about osteoporosis amongst the participants of the study was limited. Only 8.0% of the participants achieved a good score on the knowledge questionnaire. The mean and median scores of our investigation out of 100 were 33.2 ± 8 and 34 respectively. A study conducted on female medical school entrants in Sri Lanka had similar results with mean and median scores being 34.8 ± 10 and 35 respectively out of a total score out of 100 [18].

A knowledge of risk factors is imperative because the level of knowledge can be used as a tool to inform prevention programs for the disease [13]. Nevertheless, knowledge of risk factors amongst the participants was poor when compared to the college females in US in a similar study [19]. Previous studies conducted in Pakistan have shown that women do not possess a significant knowledge about the risk factors for osteoporosis and the knowledge does not necessarily translate into practicing prevention [13]. However, the study had a higher mean score on knowledge of osteoporosis as compared to a similar study conducted amongst university students in Quetta where the average knowledge score was 13.01 ± 2.9 [10]. This is indicative of regional disparity in knowledge about the disease.

The Health Belief Model suggests preventative measures will be adopted by participants only if they feel susceptible to the disease and if they perceive that the seriousness of the disease will considerably affect their lives [20]. The perceived susceptibility towards osteoporosis in our study was low with only 14.0% of participants believing that they could develop osteoporosis. However, more than half of the participants believed that osteoporosis is a serious disease, while more than three quarters of them considered it to be a barrier in their daily routines. These results coincide with a study conducted amongst US college students where perceived susceptibility was low but perceived seriousness was high [21]. Although participants reported barriers towards calcium intake, a much larger proportion of participants were positive about the benefits of a calcium rich diet.

Studies conducted in Pakistan have shown inadequate levels of exercise and an inactive lifestyle is on the rise which maybe a result of increased indoor hours [22]. The absence of required levels of exercise can play an important role in contributing towards low bone density and muscle atrophy both of which increase the risk for osteoporosis [23]. Our study showed that exercise types and durations were way below par with only 12.0% of the population engaging in exercise of the recommended type and duration (>90 min). This does not match the level of motivation towards exercise which was expressed, as results showed that 22.8% of the participants were motivated to exercise regularly. The commonest form of exercise was walking and majority of the participants (43.0%) engaged in exercise for less than 30 min per week. This is similar to a study conducted amongst women in New Zealand where the most popular form of exercise was also walking (42.0% in age groups 20–29 years) and majority of the population engaged in exercise 20–30 min per week across all age groups selected for the study [24].

Adequate calcium intake is necessary to reduce the risk of osteoporosis. Sufficient nutritional intake of calcium is advised as one of the preventative methods for osteoporosis [7]. Our study revealed that only 29.0% of the participants attained the RDA for calcium although they were motivated towards consuming a calcium rich diet. The average calcium consumption of our study subjects was 510 mg/day. This was similar to a study conducted amongst Iranian women, in which most subjects (50.9%) had calcium intake lesser than 60% of the recommended daily intake (1000 mg/day) [25].

The majority of the study participants were unaware of the role of smoking as a risk factor for osteoporosis. 15.0% though that it could lead to the disease. In another study conducted amongst Pakistani women, 42.76% of participants did not know about the role of smoking in osteoporosis [4]. This reflects that the lack of knowledge with regard to this aspect extends from a specific population (medical school entrants, in this case) to the general population as a whole.

The results of our study reflect that were no significant differences amongst the participants from the two universities in most of the categories. However, students of DUHS were better aware of the lack of exercise being a risk factor for osteoporosis as well as family history making a person more susceptible to the disease. On the other hand, students of JSMU were better informed with regard to the sources for calcium intake.

There are some limitations to the study. Only two medical schools were included so the population studied was limited. Knowledge about osteoporosis was assessed through identification of possible risk factors. This format might have encouraged guessing and might not be a true reflection of the participants’ knowledge about osteoporosis. Moreover, further factors such as socio-economic discrepancies, access to exercise facilities and healthy diet were not explored in the study. These factors could have been the reason for differences noted amongst the two study populations. Further research specifically targeted to assess these parameters would be useful in understanding the noted differences.

This study reflects the need about not only spreading awareness about osteoporosis among young females but also for promoting preventative practices for the disease. This would include educating people about calcium intake and physical activity.

## Conclusion

It can be concluded that the participants had a limited knowledge about osteoporosis. Knowledge did not translate into practices for preventing osteoporosis. Consequently, there is a need for health education amongst Pakistani females pertaining to beliefs and practices about osteoporosis in order to reduce the burden of disease on the community.

## Abbreviations

DUHS: DOW University of Health Sciences
JSMU: Jinnah Sindh Medical University
OKAT: Osteoporosis Knowledge Assessment Tool
OHBS: Osteoporosis Health Belief Scale
RDA: Recommended Dietary Allowance
